# Prosecution

**Published:** 1899-06

**Authors:** 


### EDITORIAL.

### Prosecution.

The following is interesting to all dental college faculties. It is a little difficult to see or imagine upon what grounds this suit was brought, as the alleged ground was very sandy, and quick sand at that. Were such prosecutions sustained, the dental colleges had better close their doors :

In the case of Henrietta Christianson, whose suit for $25,000 damages against the North-western University Dental School, for refusing to graduate her and grant a diploma as doctor of dental surgery after she had spent three years in studying, was heard yesterday. Judge Chytraus has decided that a recovery by the plaintiff is precluded on the allegations set forth unless malice on the part of the directors and faculty of the college is proved. The court held that it was not the province of a jury or judge to decide whether a person was qualified to be graduated from an institution, unless it was shown that malice entered into the case presented.

Miss Christianson did not prove that there was any malice on the part of the defendant officers of the college and the defense set forth that the plaintiff had failed to pass in four of her studies and that was the reason for her being refused a diploma. The plaintiff, at the trial, alleged that she had spent in the neighborhood of $200 in purchasing a wardrobe for the graduation exercises in which she had been led to believe that she would participate.

When the court's decision was announced the plaintiff's attorney took a non-suit in the case and the jury was discharged.

